# Visualized Study on a New Preformed Particle Gels (PPG) + Polymer System to Enhance Oil Recovery by Oil Saturation Monitoring Online Flooding Experiment

**DOI:** 10.3390/gels9020081

**Published:** 2023-01-18

**Authors:** Yanfu Pi, Jinxin Liu, Ruibo Cao, Li Liu, Yingxue Ma, Xinyang Gu, Xianjie Li, Xinyu Fan, Mingjia Zhao

**Affiliations:** 1 Key Laboratory of Enhanced Oil and Gas Recovery of Ministry of Education, Northeast Petroleum University, Daqing 163319, China; 2 Enhanced Oil Recovery Laboratory, Exploration and Development Research Institute, Daqing Oilfield Company Limited, Daqing 163712, China

**Keywords:** flooding system of PPG + polymer, oil saturation monitoring device, enhanced oil recovery, large-scale 3D physical model, performance evaluation

## Abstract

After tertiary recovery from the oilfields, improving the production of the remaining hydrocarbon is always challenging. To significantly improve oil recovery, a heterogeneous composite flooding system has been developed with preformed particle gels (PPG) and polymers according to the technical approach of plugging and flooding combination. In addition, an oil saturation monitoring device and a large-scale 3D physical model were designed to better evaluate the performance of the technique. The evaluation results show that the viscosity, stability, and elasticity of the heterogeneous composite flooding system are better than the single polymer system. In addition, both systems exhibit pseudoplastic fluid characteristics and follow the principle of shear thinning. The results of seepage experiments showed that PPG migrates alternately in porous media in the manner of “piling plugging-pressure increasing-deformation migration”. The heterogeneous composite system can migrate to the depths of the oil layer, which improves the injection profile. In the visualization experiment, the heterogeneous composite system preferentially flowed into the high-permeability layer, which increased the seepage resistance and forced the subsequent fluid to flow into the medium and low permeability layers. The average saturation of the high, medium, and low permeability layers decreased by 4.74%, 9.51%, and 17.12%, respectively, and the recovery factor was further improved by 13.56% after the polymer flooding.

## 1. Introduction

Daqing Oilfield is the largest continental sandstone oilfield in China. Since the large-scale application of polymer flooding technology in 1996, it has produced more than 10 million tons of oil annually for more than 20 consecutive years, making a considerable contribution to ensuring the country’s energy supply [1,2,3]. As of 2021, most of the major oil layers in Daqing Oilfield have entered the subsequent water flooding stage of polymer flooding. The oil recovery was as high as 57%, and more than 40% of the geological reserves remained underground. It is urgent to explore alternative technologies. However, after polymer flooding, the oil layer is scoured by injected water and polymer for a long time, forming a dominant seepage channel characterized by low residual oil saturation and high permeability. This can easily lead to the inefficient or ineffective circulation of the displacement medium [4,5]. Therefore, the alternative technology should be able to achieve control of the dominant seepage channel and further improve the recovery factor significantly.

Most experts and scholars have performed extensive investigations on enhanced oil recovery technologies after polymer flooding [6,7,8,9,10]. After years of development, polymer-centered profile control technologies have been developed, such as high-concentration polymer flooding technology, weak gel flooding technology, and preformed gel particle technology [11,12,13]. The dominant seepage channels are blocked to expand the swept volume and improve the oil recovery. Although this technology has achieved good results in the test of Daqing Oilfield with an oil recovery increment of 8%, there exists the problem of low economic benefit due to extensive polymer consumption [14,15]. The core of weak gel technology is the cross-linking reaction between polymers and cross-linking agents underground [16,17,18]. Due to the controllable gel formation time, the reaction can form weak gels at the designated position to achieve the effect of deep profile control [19]. Some scholars have pointed out that the system may not form gels in the far-well zone under the impact of adsorption and retention of cross-linking agents and polymers during the migration in the porous media [20]. The increase in injection pressure may be caused by gel plugging near the well. Therefore, PPG technology was invented to overcome the limitations of gel technology [21]. The polymers and the cross-linking agents undergo a cross-linking reaction to form a three-dimensional gel network with a large number of hydrophilic groups [22]. After drying, pulverizing, granulating, sieving, and other technological processes, gel particles are prepared for the actual reservoir pore size [23,24]. The gel particles and the polymer solution form a heterogeneous system with the combined effects of plugging and flooding [25]. At present, gel particles are generally prepared by copolymerizing methylenebisacrylamide (a cross-linking monomer) with acrylamide [26,27]. Because there is only one methylene group in the cross-linking monomer, the gel particles have poor elasticity and are easily broken [28]. Thus, it is challenging to possess a profound blocking effect. Daqing Oilfield has independently developed new gel particles by using 4–6 alkylene bisacrylamide to copolymerize with acrylamide and acrylic acid, and the elasticity of gel particles can be significantly improved [29]. After the successful development of particles, it is urgent to carry out relevant performance evaluation experiments to gain an overall understanding of the performance of these particles.

With the development of gel technology, the relevant performance evaluation methods have also been continuously modified and updated [30,31]. With the development of gel technology, the relevant performance evaluation methods have also been continuously modified and updated. In recent years, visualization technology has been gradually applied to the study of polymer gel systems with its unique analytical advantages. At present, the main visualization technologies include etched model technology, nuclear magnetic resonance (NMR) technology and CT technology [28,32,33,34]. Observing the behavior of PPG in the etched model is one of the popular methods to evaluate the plugging effect. However, due to the fact that this technology mainly relies on optical microscopes, the etched model is required to have a certain transmittance. This means that the thickness of the model should be less than 3 mm. Wang Lihui [28] used the etched model to reveal the migration law of PPG. The length, width, and height of the model were 63, 63, and 3 mm, respectively. In NMR and CT technology, visualization equipment is coupled with oil displacement device to produce images of fluid saturation distribution and inaccessible pore volume (IPV) in real time. Shehzad Ahmed imaged [34] the profile of cylindrical core with a length of 15.24 cm and a diameter of 3.81 cm by CT technology. The plugging effect of oil displacement system was evaluated. Similarly, Wanli Kang [35] studied the distribution of residual oil at different stages by NMR technology. The length and diameter of cores in his research were 30 cm and 2.5 cm, respectively.

In the above researches, due to the limitation of visualization equipment and core holder size, the cores dimension were one-dimensional. However, the main contradiction is interlayer contradiction after polymer flooding. The cores and etching model only reflected the in-layer oil displacement efficiency. The improvement effect of the interlayer profile is not clear. Therefore, the author developed a visualization device for oil saturation monitoring device and a large-scale three-dimensional physical model without a core holder, which is free of space constraints. In this paper, the static properties (viscosity, stability, and rheological properties) of the PPG/polymer system were first evaluated. Secondly, the modified core model was utilized to test the seepage performance. Finally, the oil displacement experiment was conducted through the self-developed oil saturation monitoring device and a large-scale three-dimensional physical model to evaluate the oil displacement effect. The experimental results are essential for the popularization and application of this system to improve oil production in similar fields.

The structure of this paper is not common. Follow the order of introduction, results and discussion, methods, conclusions, and references.

## 2. Results and Discussion

### 2.1. Performance Evaluation

#### 2.1.1. Evaluation of Viscosity Performance

**Figure 1:**
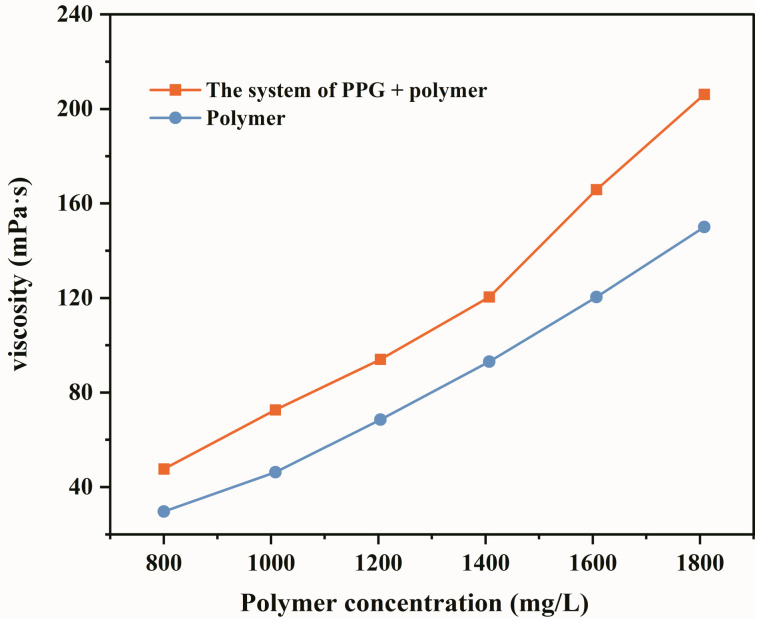
Viscosity curves of the solution of PPG + polymer and polymer solution.

After polymer flooding in Daqing Oilfield, the heterogeneity of the reservoir is enhanced, and the original mid-point polymer solution tends to migrate along the high-permeability layer, resulting in a low degree of production of the medium-low permeability layer, which affects the overall enhanced oil recovery effect [36]. According to the technical approach of combined plugging and flooding effects, the relative molecular weight and concentration of the polymer were increased, and a heterogeneous composite flooding system was formed (PPG + polymer). In order to evaluate the effect of increasing the viscosity, a Brookfield-II viscometer was used to measure the viscosity of the system before and after adding PPG. The experimental results are presented in Figure 1. As shown in Figure 1, the viscosity of the system after adding PPG is nearly 30% higher than that of the single polymer solution, indicating that the PPG particles have an apparent viscosifying effect. The increase in viscosity is mainly caused by increasing intramolecular friction. PPG particles with the viscoelastic feature form dispersions when added to a polymer solution. Among them, PPG is the dispersed phase, and the polymer solution is the dispersion medium. The intermolecular friction force includes PPG intermolecular friction force, polymer intermolecular friction force, and PPG molecule and polymer molecule internal friction force. The viscosity of the PPG-polymer system increases as the intermolecular interactions enhance. After the implementation of high concentration polymer flooding technology in Daqing Oilfield, there are obvious economic benefits [29]. The viscosity increasing effect of PPG suggests that when the viscosity of the two systems is the same, PPG can reduce the amount of polymer and further improve the economic benefits. Qian Gao [29] also evaluated the viscosity increasing performance of PPG + alkaline-surfactant-polymer (ASP) system, and reached a similar conclusion that the viscosity of the heterogeneous composite flooding system is nearly 25% higher than that of ASP. Therefore, from the perspective of economic benefits, there is a good development prospect for PPG + polymer system.

#### 2.1.2. Evaluation of Viscosity Stability

**Figure 2:**
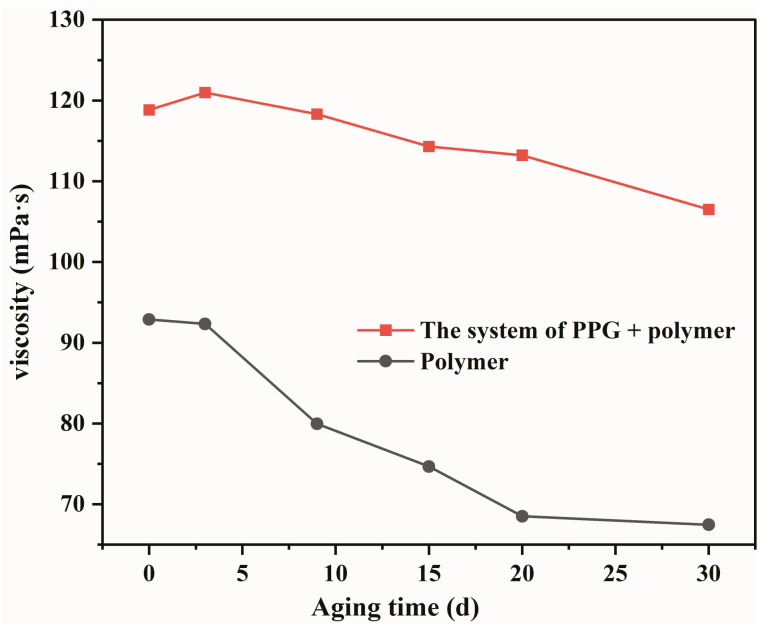
Viscosity stability curves of the system of PPG + polymer.

**Figure 3:**
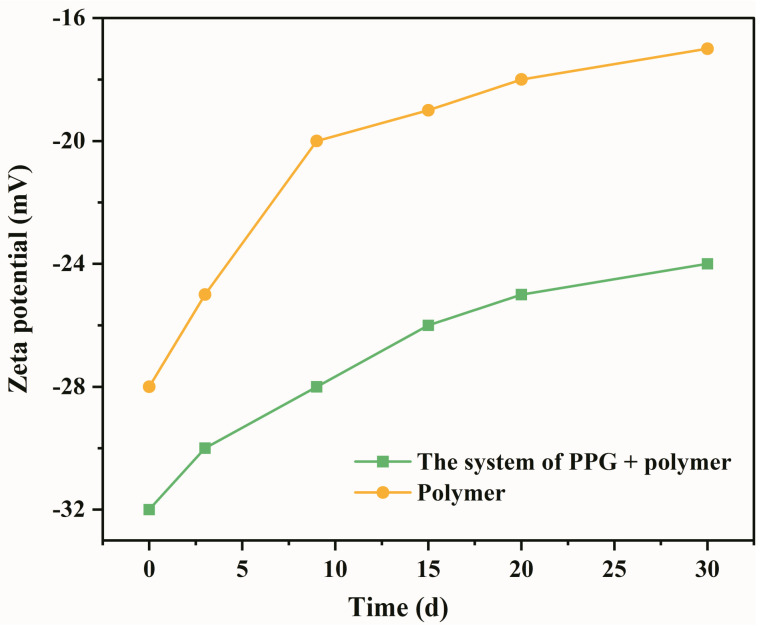
Zeta potential of two different flooding systems: Polymer and the system of PPG + polymer.

Maintaining high viscosity at the reservoir temperature is one of the critical evaluation indicators of the heterogeneous composite system [31]. Zeta potential and viscosity change curves of the heterogeneous composite system were obtained using Mastersizer 2000 Zeta potential analyzer and Brookfield-II viscometer, and the viscosity stability of the system was evaluated based on the measurements. As shown in Figure 2, the viscosity retention rate of the heterogeneous composite system is 89.83%, which was significantly higher than the polymer system with a 72.82% viscosity retention rate. Qian Gao [29] evaluated the viscosity stability of ASP system in Daqing Oilfield. The concentrations of polymer, alkaline, and surfactant were 1400, 1200, and 1300 mg/L, respectively. The viscosity retention rate of ASP system was 68.58% within 30 days, which further reflected the advantages of the heterogeneous composite system. This observation indicates that the viscosity stability of the PPG/polymer system is stronger than the polymer system. Zeta potential is an important parameter to measure the stability of the dispersion system [13]. Higher absolute Zeta potential value results in a more stable system. In Figure 3, the Zeta potential of the PPG/polymer system is significantly higher than the polymer system, confirming that the viscosity stability of the PPG/polymer system is stronger than the polymer system.

Zeta potential in a polymer solution is mainly caused by the electrostatic repulsion between polyacrylamide molecules. Over time, Na ^+^, Ca ^2+^, and Mg ^2+^ metal ions in the prepared solution neutralize the negative charge on the polyacrylamide molecular chain, which weakens the intermolecular electrostatic repulsion. Thus, the viscosity of the polymer solution decreases. PPG is comprised of polymer monomers and cross-linking agents, which have more charged groups than polyacrylamide molecules. The electrostatic repulsion between PPG and polymer molecules increases after adding PPG to the polymer solution. The total electrostatic repulsion increases, hindering the aggregation between particles and resulting in a more stable system.

#### 2.1.3. Evaluation of Rheological Properties

**Figure 4:**
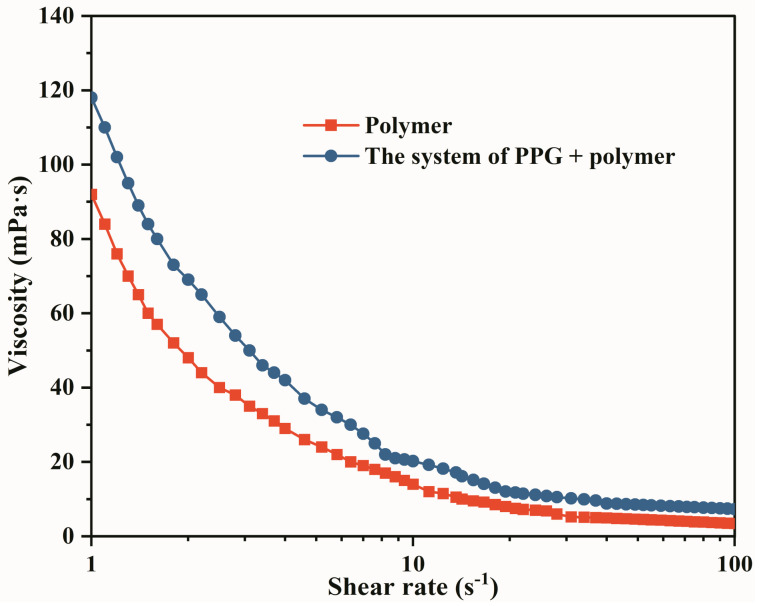
Rheological curve of the system of PPG + polymer.

During the migration process in the reservoir porous media, the oil displacement system is often subjected to the shearing effect of pore throats [23]. Under shear, changes in the fluid viscosity determine its flow behavior. Therefore, it is essential to evaluate rheological properties. In this paper, the Haake RS6000 rotary rheometer was used to test the rheology of the PPG/polymer system. The experimental results are shown in Figure 4. It can be seen from the figure that both the heterogeneous composite system and the polymer system are pseudoplastic fluids in that the viscosity decreased with the increase in the shear rate. However, the shear resistance of heterogeneous composite flooding system is stronger than that of polymer system. A similar phenomenon also occurred in another study [25]. According to the analysis, due to the branched chain of PPG molecule expanded in the polymer solution, the multiple pairs of charged groups on it increased the intermolecular interaction. It formed a more complex spatial network structure, resulting in a stronger viscosity of PPG + polymer system than a single polymer system.

#### 2.1.4. Viscoelasticity Evaluation

**Figure 5:**
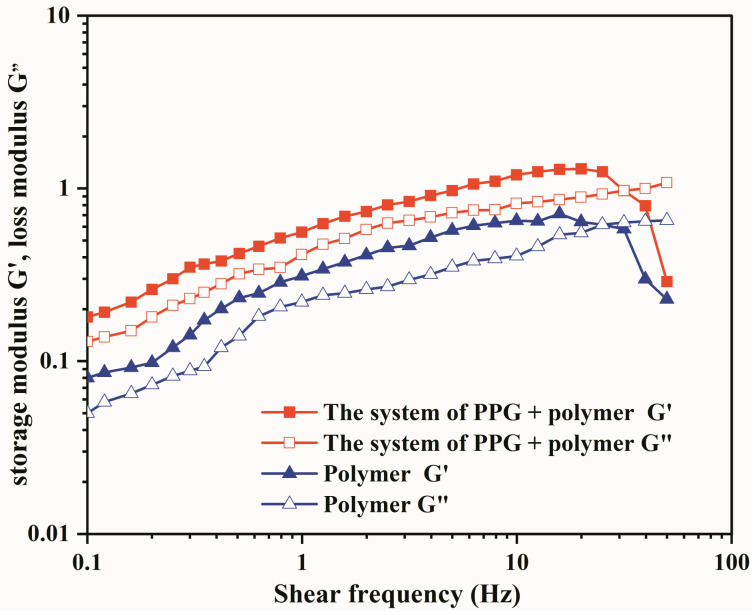
The curve of viscoelasticity in the system of PPG + polymer.

The elasticity and viscosity of the PPG and polymer system are the most important indicators to determine whether fluids can migrate to the deep layer of the oil reservoir [37]. The key parameters to quantitatively characterize elasticity and viscosity are storage modulus G′ and loss modulus G″, respectively. The curves of the above parameters with shear frequency were obtained by Haake RS6000 rotary rheometer to evaluate the viscoelasticity of PPG and polymer system. As shown in Figure 5, with the oscillation frequency increases, the elastic modulus G′ of the heterogeneous composite system and the polymer solution is higher than the viscous modulus G″. The two systems mainly exhibited elastic responses. In addition, the G′ and G″ of the heterogeneous composite system are higher than those of the polymer solution, indicating that PPG particles have the effect of increasing viscosity and elasticity. Li Wang [28] compared the elasticity factors of PPG in this research and traditional PPG. The above results were reflected from another perspective. Due to two pre-crosslinked acrylamide head groups connected by flexible chain segments in the PPG molecule, the molecular aggregate was not easy to break. Therefore, the viscoelasticity of heterogeneous composite flooding system is higher than that of polymer system.

### 2.2. Seepage Law and Oil Displacement Effect

Next, the seepage law and oil displacement effect of the system of PPG + polymer will be tested and discussed.

#### 2.2.1. Seepage Law

**Figure 6:**
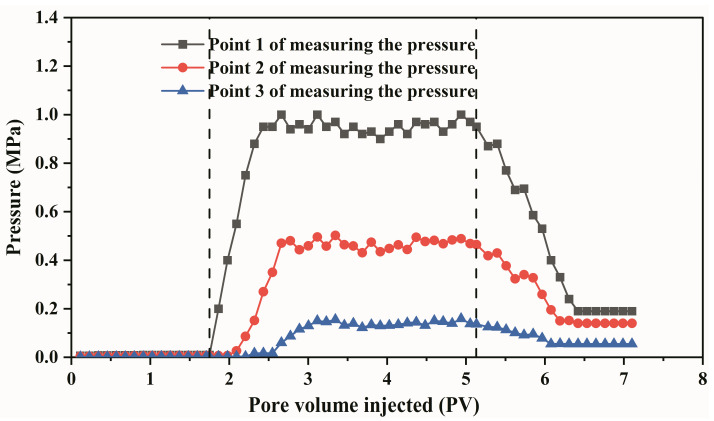
Curve of the variations of pressure at each point of measuring pressure with multiple times of injected pore volume.

As a new type of deep liquid flow diverting agent, the PPG/polymer system is expected to become the alternative technology after polymer flooding treatment in Daqing Oilfield. Whether the system of PPG + polymer possesses a deep profile control effect is determined by its seepage behavior in the porous medium. The flow experiment was carried out in the elongated core with the selected pressure measurement locations to obtain the pressure distribution curve along the core and evaluate the seepage performance of the system. The experimental results are shown in Figure 6. It can be seen from the figure that the steady pressure of each point of measuring pressure decreases successively, which are 0.9 MPa, 0.54 MPa, and 0.25 MPa, respectively. The corresponding pressure gradients are 1.08 MPa/m, 0.89 MPa/m, and 0.76 MPa/m, respectively. The resistance coefficient (*F*_r_) and the residual resistance coefficient (*F*_rr_) are often used to evaluate the seepage ability of fluids in porous media [38]. Their values can be obtained through these experiments and the corresponding calculation formulas as follows:

(1) $$F_{r}=\frac{\Delta p_{2}}{\Delta p_{1}}$$

where △*p*_1_ is the stable pressure difference in the water flooding stage in MPa; △*p*_2_ is the stable pressure difference in the regulating flooding stage in MPa.

(2) $$F_{\mathrm{rr}}=\frac{\Delta p_{3}}{\Delta p_{1}}$$

where △*p*_3_ is the stable pressure difference in the subsequent water flooding stage in MPa.

**Figure 7:**
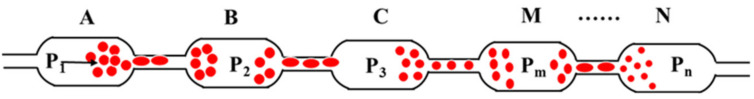
Schematic diagram of PPG accumulation and migration process in core pore throats.

The resistance coefficient and residual resistance coefficient obtained from Formulas (1) and (2) are 50 and 21, respectively. The results indicate that the PPG/polymer system can migrate to the deep layer of the core and possess the effect of deep profile control. Ali K. Alhuraishawy [38] synthesized PPG with traditional N, N’- methylenebisacrylamide, acrylamide, and acrylic acid. The plugging ability of PPG + low salinity water system was evaluated by the two-dimensional model with the residual resistance coefficient. When the salinity of injected brine is 0.01%, the Frr was only 2. The residual resistance coefficient of the heterogeneous system in this paper is nearly 10 times that of the system, which reflects the advantages of this system. The PPG particles swell with water and migrate in the core, which can be simplified as the process shown in Figure 7. PPG particles are naturally selected by the size of pore throats and accumulate at smaller throats when flowing through porous media, resulting in a “pressure superposition effect” and a continuous increase in the injection pressure. When the injection pressure reaches the start-up pressure of PPG, the particles deform under the action of external force, break through the pore throat and reduce the pressure. The PPG particles migrate alternately in the porous medium in the manner of “piling plugging-pressure increasing-deformation migration”. The particle size decreased due to the PPG and polymer shape rearrangement under the action of the core shear. Combined with the adsorption and retention of the polymer solution in the core, the pressure gradient along the process decreases with the increase in injection distance.

#### 2.2.2. Displacement Characteristics

(1) Resistance–oil saturation relationship curve

**Figure 8:**
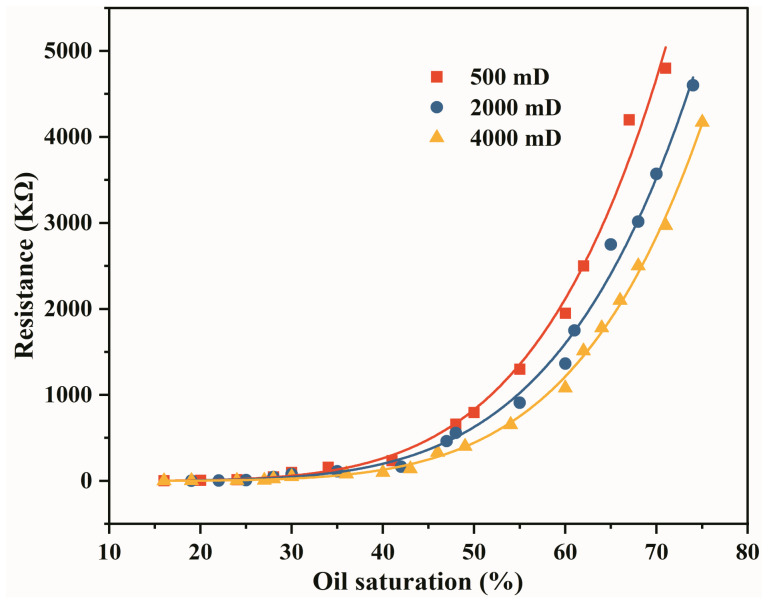
Relationship between resistance and oil saturation under different permeability conditions.

In order to complete the conversion of resistance to oil saturation in the displacement experiment of the large-scale 3D core model, the resistance–oil saturation relationship curve was obtained by standard cores. As shown in Figure 8, the resistance value under different permeability increases with the increase in oil saturation, indicating a clear correlation between the electrical resistance value and the oil saturation [39,40]. The dynamic monitoring of oil saturation during oil displacement can be achieved from the curves.

(2) Oil displacement effect

**Figure 9:**
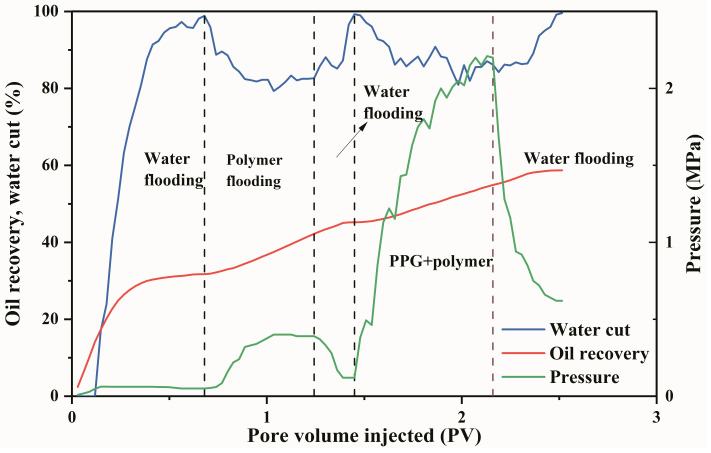
Variation curves of each parameter with the injection pore volume.

A large-scale 3D physical model was used to perform displacement experiments to evaluate the oil displacement effect of the PPG/polymer system. The experimental results are shown in Figure 9 and Table 1. The oil recovery after polymer flooding is 46.2%, and 54.8% of the oil has still remained in the reservoir. After injecting 0.7 PV of PPG + polymer mixture, the oil recovery was further increased by 13.56%. The oil displacement features of the system of PPG + polymer can be analyzed from the water cut and pressure changes. During the injection of the PPG + polymer mixture, the injection pressure increased in a zigzag manner, reaching a maximum of 2.2 MPa. As the pressure increased, the water cut decreased by approximately 20% and was relatively constant for a long time, indicating that the system of PPG + polymer established a stable displacement pressure system. In addition, changes in pressure were related to changes in the seepage field. The system of PPG + polymer expanded the swept volume and effectively displaces the remaining oil that cannot be swept by the polymer flooding. Thus, the PPG + polymer system can decrease the water cut and substantially enhance oil recovery.

(3) Pressure and remaining oil saturation

**Figure 10:**
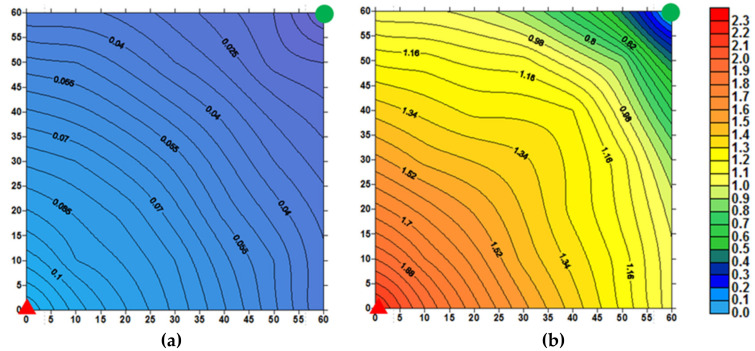
Distribution of core pressure field before and after PPG/polymer flooding injection. (**a**) Distribution of core pressure field before PPG + polymer flooding. (**b**) Distribution of core pressure field at the end of PPG + polymer flooding.

Combined with the changes in the plane pressure field and remaining oil saturation field before and after the PPG + polymer flooding, the depletion degree of each layer was further analyzed. In Figure 10, the pressure gradually decreases from the injection well to the production well, and the pressure gradient near the injection well is larger than that of the production well. In the PPG + polymer flooding stage, the PPG and polymer solution increase the overall pressure inside the model, and a significant pressure drop occurs near the injection well. The pressure changes indicate that the PPG particles and the polymer solution enhance the seepage resistance, leading to an increase in injection pressure. However, due to the shearing effect of the porous medium and the adsorption and retention of the polymer solution, the pressure gradually decreases along the direction from injection to the production end.

**Figure 11:**
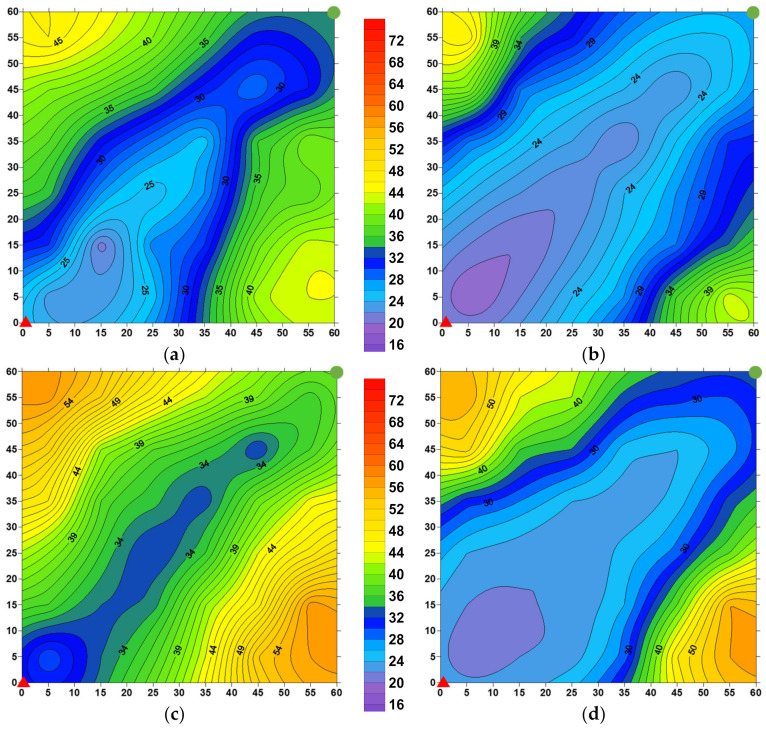

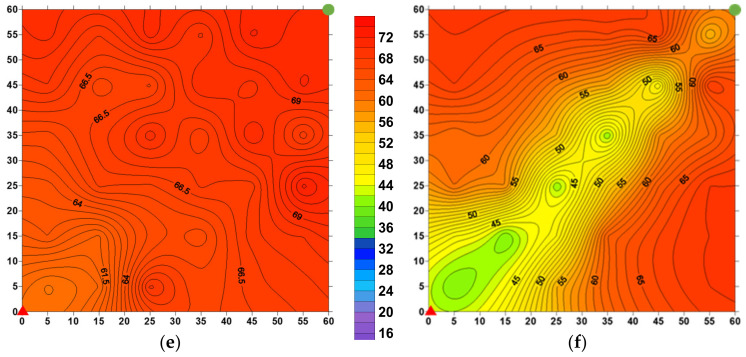
Distribution of core saturation field before and after PPG/polymer flooding injection. (**a**) Saturation field of high permeability layer at the end of polymer flooding; (**b**) Saturation field after injection of high permeability layer; (**c**) Saturation field of medium permeability layer at the end of polymer flooding; (**d**) Saturation field after PPG/polymer injection in the middle permeability layer; (**e**) Saturation field of low permeability layer at the end of polymer flooding; (**f**) Saturation field after PPG/polymer injection in low permeability layer.

The oil saturation map reflects the depletion degree of each layer, which can be used to analyze the oil displacement features of applying the PPG/polymer system. As shown in Figure 11, the red color represents areas with high oil saturation of 54–74%, the green and yellow colors represent areas with medium oil saturation (34–54%), and the blue color represents areas with low oil saturation (0~34%). The grid method is utilized to calculate the sweep coefficient and oil displacement efficiency of the high, medium, and low layers. The calculation results are shown in Table 2.

From the saturation field map after polymer flooding, low and medium oil saturation bands appear in the layers with medium and high permeability from the injection well to the production well. However, all points in the low permeability layer have high oil saturation. After completing the polymer flooding, the average saturation of each layer is 33.09%, 41.69%, and 66.7%, and the recovery degree is 57.72%, 43.19%, and 2.4%, respectively (as shown in Table 2). The polymer solution primarily entered the high and medium permeability layer during polymer flooding, which formed a main flow channel between the injection and production wells. Therefore, the oil saturation decreases significantly in the main flow area. The oil layer is not entirely produced due to less liquid absorbed in the low permeability layer. In summary, the liquid absorption intensity directly impacts the displacement effect. The oil displacement efficiencies of the high, medium, and low permeability layers were 72.12%, 68.76%, and 52.08%, respectively. In addition, the sweep coefficients are 80.03%, 62.8%, and 4.6%, respectively (Table 2). The remaining oil after the polymer flooding is mainly located in the layers with medium and low permeability, which has a more substantial tapping potential.

After switching to PPG/polymer flooding, the low-saturation zone in the high permeability layer is further extended, and the saturation of the mainstream channel in the medium permeability layers decreases. At the same time, a medium saturation band appeared between the injection and production wells in the low permeability layer. The average saturation of the high, medium, and low permeability layers decreases by 4.74%, 9.51%, and 17.12%, and the oil recovery increases by 6.06%, 12.96%, and 25.05%, respectively (Table 2). The difference in saturation drop indicates that the PPG/polymer system mainly enhances oil recovery in the layers with medium and low permeability. In the PPG/polymer flooding stage, the PPG/polymer preferentially enters the hyperpermeable layer due to the impact of heterogeneity. Among them, the PPG swells in contact with water and blocks the main flow channel. Subsequently, the fluid after PPG was forced to divert, increasing liquid absorption in the medium and low permeability layers. The high viscosity of the polymer solution can significantly improve the oil displacement efficiency. After the PPG + polymer flooding, the oil displacement efficiency of the high, medium, and low permeability layers is increased by 2.72%, 4.03%, and 18.87%, and the sweep coefficient is increased by 5.18%, 14.33%, and 35.09%, respectively (Table 2). In summary, the PPG/polymer system combines the effects of plugging and flooding, which can further greatly improve the recovery factor after polymer flooding.

A new type of PPG has been developed in this paper, which shows excellent performance. There are broad development prospects for this PPG. The application of gel in reservoir development has been promoted to a certain extent. In addition, a saturation monitoring device and a large three-dimensional physical model have also been developed. Compared with NMR [35] and CT [34] technology, the law of PPG longitudinal interlayer profile control can be more revealed by saturation monitoring device. However, it is worth noting that this device is still unable to achieve the recognition accuracy of the pore throat scale.

## 3. Conclusions

This study investigates the challenging technical issue of greatly enhancing oil recovery after polymer flooding. A heterogeneous system consisting of PPG particles and polymers independently was developed for Daqing Oilfield, and the basic performance of the system was systematically evaluated. Subsequently, the physical model and the device for monitoring saturation were used to evaluate the seepage performance and oil displacement effect of the system. In the end, the conclusions are summarized as follows:

(1) PPG particles possess viscosifying and elastic properties. The viscosity of the heterogeneous composite system is 30% higher than that of the single polymer solution, and the elastic modulus G′ and loss modulus G″ are significantly improved by adding PPG particles.
(2) The viscosity retention rate of the heterogeneous composite system within 30 days is 90%, which is 17% higher than that of the polymer system.
(3) PPG migrates alternately in porous media in the manner of “piling plugging-pressure increasing-deformation migration”. After compounding with polymer, the composite system can migrate to deep oil layers and act as plugging. The pressure gradients along the long core were 1.08 MPa/m, 0.89 MPa/m, and 0.76 MPa/m, respectively, and the pressure gradient decreased slightly. The resistance coefficient and residual resistance coefficient are 50 and 21, respectively. In large-scale 3D heterogeneous cores, the composite system appears to preferentially enter the high-permeability layer, which increases the seepage resistance and forces the subsequent fluids to enter the medium/low permeability layers, thereby greatly enhancing oil recovery in the medium/low permeability layers.
(4) The heterogeneous composite system enhanced oil recovery by 13.56% after polymer flooding. The average saturation of high, medium, and low permeability layers is decreased by 4.74%, 9.51, and 17.12%, respectively, and the oil displacement efficiency was increased by 2.72%, 4.03%, and 18.87%, respectively. The sweep coefficients were increased by 5.18%, 14.33%, and 34.09%, respectively, and the medium/low permeability layers were produced on a large scale.

## 4. Materials and Methods

### 4.1. Experimental Materials

Four primary experiment materials were needed in this study: water, simulated oil, polymer, and PPG, which will be discussed as follows.

Water: According to the ion composition of formation water in Daqing Oilfield, the simulated formation water with a salinity of 6778 mg/L was prepared. The simulated formation water was mainly used in the core flooding experiment. The reagents and contents used in this study are shown in Table 3. Sewage and clean water provided by Daqing Oilfield were used to prepare polymer and PPG solutions.

Simulated oil: The oil and aviation kerosene of Daqing Oilfield were mixed in a ratio a ratio of 3:1, and the viscosity was 9.8 mPa·s.

Polymer: Polyacrylamide was produced by a refining company in Daqing, with a relative molecular mass of 2500 × 10^4^, and the solid content was 91%.

**Figure 12:**
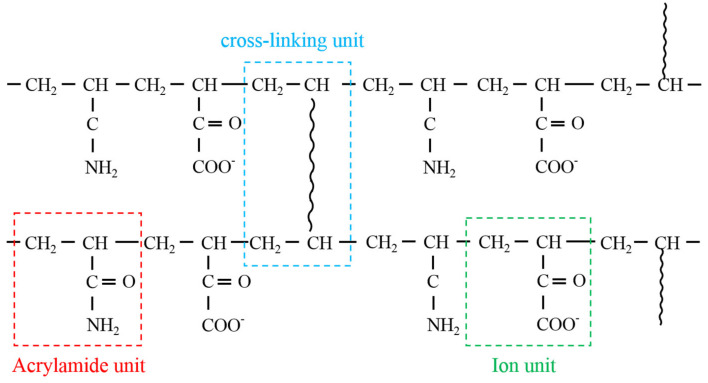
Schematic diagram of the molecular structure of PPG.

PPG: The expansion ratio of the particles is 3, and the particle size was between 0.15 mm and 0.3 mm. It was prepared by copolymerizing 4–6 alkylene bisacrylamide and acrylamide (Figure 12), which significantly improved the elasticity compared with traditional PPG. It was provided by Daqing Oilfield Exploration and Development Research Institute.

Due to the limitation of coring technology, it is difficult to extract large and complete natural cores from the reservoir. Therefore, the physical models used in this paper are artificial. They were prepared by pressing and cutting based on quartz sand as the matrix, epoxy resin and ethylenediamine as the binder.

**Figure 13:**
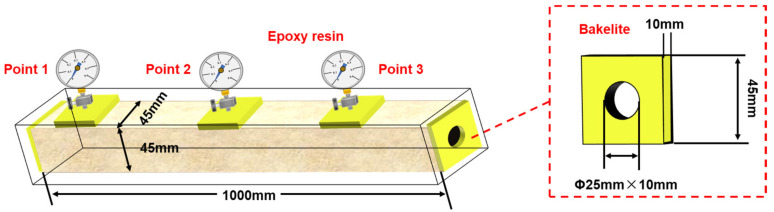
Schematic diagram of the long core model.

The physical models used in this paper mainly include a long core model and a large-scale 3D core model, which were used for the evaluation of seepage performance and oil displacement effect, respectively. Among them, the size of the artificial core model was 1000 × 45 × 45 mm (length × width × height), the permeability was 4000 × 10^−3^ μm^2^, and the porosity was 29.81%. In order to realize the flow of fluid in the model and pressure monitoring, the external surface of the model is cast with epoxy resin and bonded with high-strength bakelite with thread at different positions as the injection end, outlet end, and the pressure measuring point. Three points for measuring pressure were evenly arranged to monitor the pressure distribution of the long core model. The schematic diagram of the long core model is shown in Figure 13.

**Figure 14:**
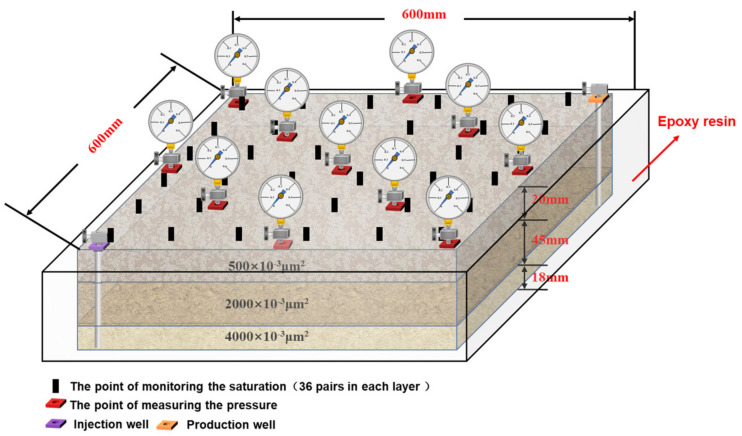
Schematic diagram of a large-scale 3D physical model.

The size of the large-scale 3D physical model was designed according to the statistical results of permeability classification and thickness ratio in the reservoir of Daqing Oilfield (Figure 14). The relevant parameters are shown in Table 4. A total of 36 pairs of electrodes are arranged in each layer of this model. The dynamic monitoring of the oil displacement process can be realized by the device monitoring oil saturation.

### 4.2. Experimental Methods

#### 4.2.1. Performance Evaluation Experiment

PPG static properties include viscosifying, stabilization, rheological, and viscoelastic properties, which were not measured in a porous medium. The performance test methods were as follows:

1. Viscosity test

The viscosity of the polymer solution and the heterogeneous composite system with different concentrations at 45 °C was measured by Brookfield-II viscometer (China, Taizhou City, Haian Petroleum Inc.) to evaluate the viscosity-increasing performance. According to the principle of matching the particle size of PPG with that of the reservoir porous medium, the particle size of PPG was designed between 0.15 mm and 0.3 mm, and the concentration was fixed at 500 mg/L. The polymer solution concentrations were 800, 1000, 1200, 1400, 1600, and 1800 mg/L, respectively. The above solutions were all prepared by diluting polymer solution with a concentration of 5000 mg/L with pure water.

The experimental steps are as follows: (1) The viscometer was turned on and rotor 61 was selected. (2) The rotor is screwed into the screw and the speed is set to 6 r/min. (3) The solution was slowly poured into the container fixed on the viscometer hook. (4) The knob that controls the raising and lowering was turned to make the rotor slowly lower until its upper surface is immersed in solution. (5) The “Start” key was pressed and the viscosity value after stabilization was recorded. (6) The container and rotor were removed in turn for cleaning after the test.

2. Viscosity stability test

The stability of the dispersion system depends on the mutual repulsion strength between dispersed phase particles. Larger strength can increase the difficulty of particle aggregation, resulting in a more stable system. Zeta potential is a measure of the strength of mutual repulsion between particles and can be used to characterize the stability of dispersion systems. Mastersizer 2000 Zeta potential analyzer and Brookfield-II viscometer (China, Shenzhen City, Yihua New Electronics Inc.) were used to measure the Zeta potential and viscosity of the heterogeneous composite system at different times to evaluate the stability of the system. In the system, the PPG concentration is 500 mg/L, and the polymer concentration was 1400 mg/L.

(1) The switch, display, and program (BIC Zeta potential Analyzer) in the Zeta potential analyzer were opened in turn. (2) The solution was poured into 1/3 of the height of the cuvette, and then the palladium electrode was inserted into the solution. (3) At the beginning of measurement, the Zeta potential value recorded after stabilization (4) The palladium electrode and cuvette were cleaned. Subsequently, the next sample was tested by above steps.

3. Rheological and viscoelastic performance experiments

The rheology and viscoelasticity of the PPG/polymer system were tested using the Haake RS6000 rotary rheometer (China, Taizhou City, Haian Petroleum Inc.), and the shear rate test range is 1~100 s^−1^. The system concentration was the same as the viscosity stability experiment.

The rheology test steps are as follows:

(1) The rheometer was turned on, and then the Rheo Win 7 Job Manager software was run. (2) The prepared solution was poured into the beaker and placed on the tray. Subsequently, the rotor was installed and the rotor type was selected in the software. (3) The position of the rheometer tray was adjusted and the test was started. (4) The beaker and rotor were cleaned. Subsequently, the next sample was tested by above steps.

#### 4.2.2. Experiment Using Seepage Law to Evaluate the Oil Displacement Effect

1. Flow experiment in the long core

A flow experiment was carried out on a core with a length of 1 m to evaluate the seepage performance of the PPG/polymer system. The system concentration was the same as the viscosity stability experiment. The experimental procedure was the following: First, water was injected into the core at a flow rate of 0.3 mL/min until the pressure was stable after the model was evacuated with a vacuum pump. Then, the PPG/polymer system was injected with the same flow rate until the pressure was stable again. Finally, water flooding was conducted at a stable pressure.

2. Displacement experiment of the large 3D physical model

The oil saturation monitoring device and the large-scale three-dimensional physical model were used to carry out oil displacement experiments, and the production curves and oil saturation maps were obtained to evaluate the oil displacement effect of the PPG/polymer system. The fundamental theory of the oil saturation device is Archie’s Formula (3):

(3) $$F=\frac{R_{o}}{R_{w}}=\frac{a}{\varphi^{m}}$$

(4) $$I=\frac{R_{t}}{R_{o}}=\frac{b}{S_{w}^{n}}$$

where *F* refers to the relative resistivity, *m* is the cementation index, which is related to pore structure and the degree of cementation, *φ* is the formation porosity, *I* is the resistivity increase rate, *R_o_* is rock resistivity of 100% saturated formation water, *R_w_* is the resistivity of the formation water in the pores, *R*_t_ refers to the resistivity of the petroliferous rock, *S_w_* is the water saturation, *n* is the formation index, and *a* and *b* are the lithologic constants related to the rock properties.

The resistances under different oil saturation can be measured by Archie’s formula. In order to realize the conversion between oil saturation and electrical resistance in the oil displacement experiment, it is necessary to obtain the corresponding curve of oil saturation and electrical resistance in each layer of the large-scale 3D physical model before the start of the experiment. It can be seen from the formula that the accuracy of the relationship curve is mainly affected by the physical properties of the core. Therefore, the conventional artificial cores (30 cm × 4.5 cm × 4.5 cm) with the same physical properties as the layers of the large 3D model were used. Subsequently, the displacement experiments were performed under the same experimental procedure. Ultimately, the standard relationship between oil saturation and resistance was obtained.

**Figure 15:**
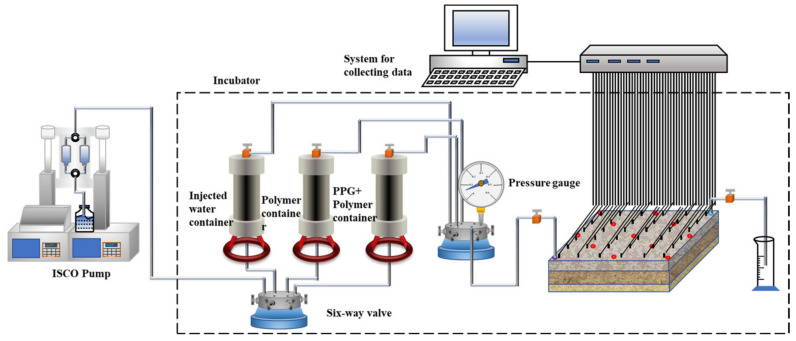
Schematic diagram of the connection of the device in the experiment of the large-scale 3D physical model.

The connection diagram of the oil displacement experimental device is shown in Figure 15. The experimental steps are as follows: after the core was saturated with oil, the water was flooded to a water cut of 98% at an injection rate of 0.5 mL/min; after injection of a 0.57 PV concentration of 1000 mg/L medium fraction polymer, the water was flooded to reach a water cut value of 98%; after 0.7 PV PPG/polymer was injected, water was flooded to achieve 98% water content.

The flow of displacement equipment is shown in Figure 1. The equipment in this experiment is the same as the flow experiment, and its purpose and source are as follows:

The displacement speed and pressure were controlled by ISCO pump (USA, NE, Lincoln, Teledyne ISCO Inc.). During the experiment, the pressure change was monitored by pressure gauges (Canada, Ottawa, OMEGA Engineering Inc.) with an accuracy of ±0.01 MPa. The produced fluids were measured by the test tube (China, Nanjing City, Haian Petroleum Inc.) with an accuracy of ±0.01 mL. To ensure that the experiment was carried out at reservoir temperature, the containers and cores were placed in a thermostat (China, Nanjing City, Haian Petroleum Inc.) with an accuracy of ±0.5 °C.

## Figures and Tables

**Table 1:** The result of the flooding experiment for the system of PPG + polymer.

Scheme Number	Oil Recovery of Water Flooding (%)	Enhanced Value of Oil Recovery of Polymer Flooding (%)	Enhanced Oil Recovery of PPG + Polymer Flooding after Polymer Flooding (%)	Total Recovery (%)
1	31.74	13.46	13.51	58.71

**Table 2:** Related parameters before and after PPG/polymer injection.

Layer		High	Medium	Low
Original oil saturation (%)		78.26	73.38	68.34
Polymer flooding	Oil displacement efficiency (%)	72.12	68.76	52.08
	Sweep factor (%)	80.03	62.8	4.6
Average oil saturation at the end of polymer flooding (%)		33.09	41.69	66.7
Oil recovery at the end of polymer flooding (%)		57.72	43.19	2.4
PPG/polymer flooding	Oil displacement efficiency (%)	74.84	72.79	70.95
	Sweep factor (%)	85.21	77.13	38.69
Average oil saturation after PPG + polymer flooding (%)		28.35	32.18	49.58
Oil recovery at the end of PPG/polymer flooding (%)		63.77	56.15	27.45
Average saturation drop (%)		4.74	9.51	17.12
Increment of oil displacement efficiency (%)		2.72	4.03	18.87
Increment of sweep factor (%)		5.18	14.33	35.09
Increment of oil recovery in a single layer (%)		6.06	12.96	25.05

**Table 3:** Reagents used and corresponding concentrations.

Reagent	NaHCO_3_	NaCl	KCl	MgSO_4_	Na_2_SO_4_	CaCl_2_
Concentration (mg/L)	2718	1789	20	62	114	64

**Table 4:** Relevant parameters of the large-scale 3D physical model.

Layer	Permeability (×10^−3^ μm^2^)	Size (mm) (Length × Width × Height)	Porosity (%)	Average Porosity (%)
Low permeability layer	500	600 × 600 × 20	22.58	26.53
Intermediate permeability layer	2000	600 × 600 × 45	27.19	
High permeability layer	4000	600 × 600 × 18	29.28	

## Data Availability

Data available on request due to restrictions e.g., privacy or ethical. The data presented in this study are available on request from the corresponding author. The data are not publicly available due to company requirements.
